# Surgical necrotizing enterocolitis risk factors in extremely preterm infants: a Korean nationwide cohort study

**DOI:** 10.1038/s41390-024-03519-3

**Published:** 2024-08-24

**Authors:** Seung Hyun Kim, Joonhyuk Son, Hyun-Kyung Park

**Affiliations:** 1 Department of Pediatrics, Hanyang University College of Medicine, Seoul, Republic of Korea; 2 Department of Pediatrics, Samsung Medical Center, Sungkyunkwan University School of Medicine, Seoul, Republic of Korea; 3 Department of Pediatric Surgery, Hanyang University College of Medicine, Seoul, Republic of Korea

## Abstract

**Background:**

The incidence of necrotizing enterocolitis (NEC) is significantly associated with gestational age (GA). This study aimed to investigate risk factors for surgically treated NEC (sNEC) in extremely preterm infants (EPIs) using nationwide cohort registry.

**Methods:**

Data were collected from 16,338 very-low-birth-weight infants registered in the Korean neonatal network. Clinical data of 5310 EPIs were retrospectively analyzed. sNEC was defined as infants with diagnosis of NEC requiring surgical treatment, who underwent surgical intervention for NEC or died before surgery. Infants were categorized into three groups based on their NEC status: infants without NEC (control), medically treated NEC (mNEC), and sNEC. These groups were matched based on GA to investigate risk factors for NEC.

**Results:**

In EPIs, small for gestational age (SGA; odds ratio 1.68, 95% confidence interval [CI], 1.17–2.36, *p* = 0.004), hypotension (1.49, 1.18–1.89, *p* = 0.001), and IVH (1.63, 1.30–2.05, *p* < 0.001) were identified as risk factors for sNEC. Complete administration of antenatal steroid reduced the risk of sNEC (0.80, 0.64–0.99, *p* = 0.044).

**Conclusion:**

Our study demonstrated that EPIs who are SGA, and experience hypotension and IVH may be at an increased risk of developing NEC requiring surgery. These groups require close attention and monitoring for any signs of surgical indications of NEC.

**Impact:**

- This nationwide cohort study aimed to identify characteristics of infants with necrotizing enterocolitis (NEC) among extremely preterm infants (EPIs) and analyze the risk factors associated with NEC requiring surgical intervention.
- Small for gestational age (SGA), hypotension, and intraventricular hemorrhage (IVH) were identified as significant risk factors for surgically treated NEC (sNEC) in EPIs. The administration of antenatal steroids decreases the risk of sNEC.
- Close attention and monitoring for EPIs with early identifiable risk factors such as SGA, hypotension, and IVH should be considered to prevent and detect sNEC early, ultimately leading to improved long-term outcomes.

## Introduction

Necrotizing enterocolitis (NEC) is a severe gastrointestinal condition with elevated morbidity and mortality rates among premature infants. The reported incidence of NEC in very-low-birth-weight (VLBW) preterm infants ranges 2–13%.^1^ Surgical treatment, including primary peritoneal drains or laparotomy, is required in 27–52% of infants diagnosed with NEC.^2–4^ The mortality rate of NEC, ranging from 20–30%, is the highest among infants requiring surgery.^5–7^ The mortality rate for surgically treated NEC (sNEC) is higher than that for medically treated NEC (mNEC), and it decreases as the gestational age (GA) increases.^8–11^ sNEC is associated with significant delays in growth and risk of neurodevelopmental impairments. Compared to infants with mNEC, these risks are higher in infants with sNEC.^12–14^ This emphasizes the importance of early prediction and prevention of progression to severe NEC requiring surgical treatment, especially in more premature infants.

Some studies have identified predictive factors for sNEC in premature infants.^15,16^ Liu et al. reported a single-center study targeting preterm infants with GA < 37 weeks, and el Manouni el Hassani et al. reported a multicenter case-control study conducted with preterm infants with GA < 30 weeks. In these studies, lower GA, absence of maternal corticosteroid administration, early onset of NEC, hemodynamically significant patent ductus arteriosus (PDA), and low serum bicarbonate level were identified as risk factors for sNEC in preterm infants. To the best of our knowledge, only few national cohort studies have targeted extremely preterm infants (EPIs, infants born < 28 weeks of gestational age).^17^

The Korean Neonatal Network (KNN) is a nationwide registration system for VLBW infants in Korea that was officially established in April 2013. Seventy-seven hospitals with neonatal intensive care unit (NICU) in Korea are a part of the KNN. The data of VLBW infants in the KNN has enabled identification of prevalence of morbidities or long-term outcomes in a large population of the country. In a previous study analyzing KNN data, we demonstrated a higher proportion of sNEC than mNEC in infants born at < 27 weeks of GA, with sNEC constituting 68.7% of all NEC cases in the EPI group.^18^

This nationwide cohort study aims to investigate the risk factors associated with sNEC in EPIs with a high risk of developing sNEC, using a VLBW infant cohort in Korea.

## Methods

### Study population

Data of VLBW infants admitted to the NICU at birth or transferred from other hospitals within 28 d of life to the 77 KNN participating hospitals are registered in the KNN registry. The KNN registry was approved by the institutional review board at each participating hospital, and informed consent was obtained from the parents of each infant at enrollment in the NICUs participating in the KNN. This study was approved by the Institutional Review Board of the Hanyang University Medical Center (2024-02-035). Data from the medical records of 16,338 VLBW infants born between January 2013 and December 2020 and registered in the KNN were collected. Infants born with severe congenital anomalies, those who died within 7d after birth, or those with no information on NEC were excluded from the study. Finally, clinical data of 14,878 VLBW infants were enrolled in this study.

**Fig. 1:**
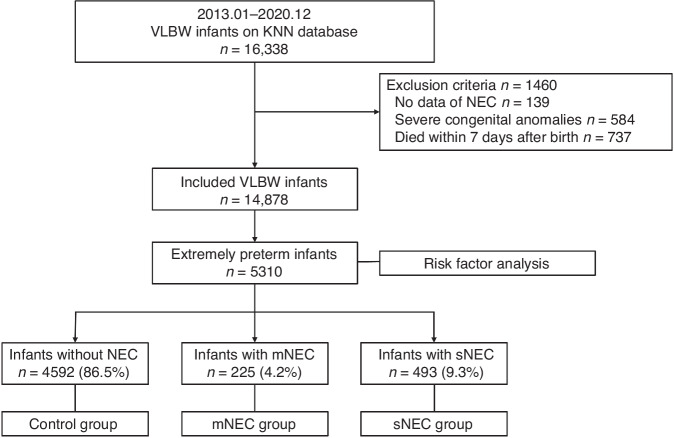
Inclusion flow chart. VLBW very-low-birth-weight; KNN Korean Neonatal Network; NEC necrotizing enterocolitis; Control group, EPIs without NEC; mNEC group, EPIs with medically treated NEC; sNEC group, EPIs with severe NEC that was treated by surgical intervention including peritoneal drainage or laparotomy, or who died before surgery with clinical evidence of requiring surgery.

For risk factor analysis of sNEC in EPIs, the clinical information of 5,310 EPIs in the KNN database was retrospectively analyzed. Infants without NEC were defined as the control group. Infants with medically treated NEC (mNEC) were included in the mNEC group. Infants who underwent surgical intervention (peritoneal drainage or laparotomy) for NEC or died before surgery with diagnosis of NEC requiring surgical treatment, were included in the sNEC group. The inclusion process is illustrated in Fig. 1.

### Definition

The definition of each disease was based on the KNN manual of operations. NEC was defined according to the modified Bell’s staging classification grade ≥ II. sNEC represents cases requiring surgical treatment for NEC and is distinguished from surgical treatment required due to spontaneous intestinal perforation. Pulmonary hypertension was determined when it was clinically suspected, confirmed by echocardiography, or detected on drug use. Respiratory distress syndrome (RDS) diagnosis was based on both clinical and radiographical findings. In this study, PDA was defined as cases with clinically significant symptoms. A diagnosis of clinically significant PDA was made if Color-flow Doppler echocardiography confirmed large left-to-right ductal flow through the PDA and if two or more of the following five criteria were present: (1) presence of a systolic murmur or continuous murmur, (2) presence of bounding pulse or hyperactive precordial pulsation, (3) difficulty in maintaining blood pressure (hypotension unresponsive to fluid therapy or dopamine treatment, with hypotension defined as below the lower limit of normal arterial blood pressure for the corrected GA), (4) worsening of respiratory status, and (5) evidence on chest radiograph (such as pulmonary congestion, and increased pulmonary blood flow accompanied by cardiomegaly). Hypotension was defined as requiring treatment with medications such as inotropics (dopamine, dobutamine, epinephrine) or hydrocortisone within 7 days after birth. Cases, where hypotension was managed without medication or treated only with volume expanders such as saline, were excluded. Intraventricular hemorrhage (IVH) was defined according to the Papile grading system grade ≥ II. Sepsis was defined as both confirmation of a positive blood culture and requirement of systemic antibiotic treatment for more than 5 days. Early-onset sepsis was defined as sepsis occurring ≤ 3 d after birth, and late-onset sepsis was defined as sepsis occurring > 3 d after birth.^19^ A low Apgar score was defined as an Apgar score < 7 measured at 1 min and 5 min after birth. The definition of bronchopulmonary dysplasia (BPD) was based on 2001 National Institute of Child Health and Human Development criteria.^20^ Small for gestational age (SGA) is defined as a birth weight below the 10th percentile for gestational age based on the Fenton preterm growth chart 2013.^21^ A complete administration of antenatal steroids was defined as the administration of systemic steroids to the mother within one week before delivery, either intramuscularly or intravenously, consisting of either two doses of betamethasone given 24 h apart or four doses of dexamethasone. The duration of oxygen therapy, noninvasive ventilation, invasive ventilation, and parenteral nutrition were measured throughout the total admission period as long-term outcomes.

### Statistical analysis

We performed analyses to identify risk factors for sNEC. Univariate analyses were performed to describe the characteristics of the study population and explore characteristics for risk factor analysis. Continuous variables were presented as median [interquartile range, IQR] and compared using the t-test or Wilcoxon rank sum test. The Shapiro-Wilk normality test was conducted to evaluate the normality of continuous variables. Categorical variables were presented as percentages and frequencies and were compared using the chi-square or Fisher’s exact test. As GA is a significantly related with incidence of NEC, GA was used as covariate for matching process. Logistic regression was used for the propensity score estimation, and a 1:5 nearest neighbor propensity score matching method was conducted. Calipers were adjusted so that the absolute value of the standardized mean difference was < 0.1. A backward stepwise logistic regression model was used to identify the risk factors for mNEC and sNEC. Variables that were significantly different (*p* < 0.05) between the infants with NEC (mNEC and sNEC) and control groups in the univariate analyses were selected as possible risk factors and were entered into the model. All statistical analyses were conducted using R software (Version 4.3.2., 2023).

## Results

### Characteristics of EPIs with NEC

A total of 5,310 EPIs were included in the risk factor analysis for NEC. The incidence of NEC was 13.5% (718/5310). Among EPIs with NEC, 225 (4.2%, 225/5310) and 493 (9.3%, 493/5310) infants were diagnosed with mNEC and sNEC, respectively. Of the infants diagnosed with sNEC, 446 underwent surgical intervention such as peritoneal drainage or laparotomy, while 47 infants died before surgery despite the need for surgical treatment. The characteristics of the EPIs with and without NEC are presented in Table 1.

**Table 1:** Characteristics of EPIs with NEC.

	Control group (*n* = 4592)	mNEC group (*n* = 225)	*p*-value	sNEC group (*n* = 493)	*p*-value
Neonatal factor					
Gestational age (weeks, median [IQR])	26.2 [25.1–27.1]	26.1 [24.9–27.1]	0.280	25.1 [24.3–26.3]	**0.000**
Birth weight (gram, median [IQR])	850 [700–990]	820 [670–990]	0.109	720 [620–870]	**0.000**
Male, *n* (%)	2394 (52.1%)	128 (56.9%)	0.185	275 (55.9%)	0.124
SGA, *n* (%)	331 (7.2%)	21 (9.3%)	0.287	52 (10.5%)	**0.010**
Multiple birth, *n* (%)	1475 (32.1%)	63 (28.0%)	0.222	168 (34.1%)	0.406
Cesarean section, *n* (%)	3375 (73.5%)	174 (77.3%)	0.231	338 (68.6%)	**0.022**
Low Apgar score at 1 min, *n* (%)	4494 (98.4%)	222 (99.1%)	0.609	461 (95.1%)	0.191
Low Apgar score at 5 min, *n* (%)	2193 (48.0%)	113 (50.4%)	0.521	284 (58.6%)	**0.000**
Resuscitation at delivery, *n* (%)	4494 (98.5%)	222 (99.1%)	0.625	482 (99.0%)	0.495
Maternal factor					
Maternal age (median [IQR])	33 [31–36]	33 [31–36]	0.701	34 [31–36]	0.751
IVF, *n* (%)	1183 (25.8%)	56 (24.9%)	0.830	123 (24.9%)	0.735
GDM, *n* (%)	395 (8.6%)	20 (8.9%)	0.978	37 (7.5%)	0.456
PIH, *n* (%)	572 (12.5%)	28 (12.4%)	1.000	58 (11.8%)	0.711
Chorioamnionitis, *n* (%)	1995 (49.8%)	92 (45.5%)	0.268	189 (47.5%)	0.408
PROM, *n* (%)	1993 (43.7%)	112 (49.8%)	0.087	213 (43.9%)	0.980
Abnormal amniotic fluid volume, *n* (%)	693 (16.5%)	29 (14.1%)	0.407	74 (17.1%)	0.831
Complete antenatal steroid, *n* (%)	2177 (56.2%)	104 (55.3%)	0.869	205 (51.6%)	0.090
Antenatal antibiotics, *n* (%)	1007 (71.2%)	49 (75.4%)	0.555	88 (66.2%)	0.263
Clinical morbidities					
Congenital infection, *n* (%)	67 (1.5%)	5 (2.2%)	0.522	9 (1.8%)	0.658
Air leak syndrome, *n* (%)	340 (7.4%)	26 (11.6%)	**0.030**	62 (12.6%)	**0.000**
Massive pulmonary hemorrhage, *n* (%)	401 (8.7%)	28 (12.4%)	0.074	79 (16.0%)	**0.000**
Pulmonary hypertension, *n* (%)	739 (16.1%)	50 (22.2%)	**0.020**	132 (26.8%)	**0.000**
RDS, *n* (%)	4463 (97.2%)	212 (94.2%)	**0.018**	483 (98.0%)	0.387
Surfactant use, *n* (%)	4497 (97.9%)	217 (96.4%)	0.204	483 (98.0%)	1.000
Symptomatic PDA, *n* (%)	2338 (50.9%)	117 (52.0%)	0.803	278 (56.4%)	**0.024**
Hypotension, *n* (%)	1924 (41.9%)	119 (52.9%)	**0.001**	323 (65.5%)	**0.000**
IVH (≥grade2), *n* (%)	1401 (30.5%)	91 (40.4%)	**0.002**	245 (49.7%)	**0.000**
Early-onset sepsis, *n* (%)	167 (3.6%)	6 (2.7%)	0.287	20 (4.1%)	0.730
Late-onset sepsis, *n* (%)	1392 (30.3%)	97 (43.1%)	**0.000**	248 (50.3%)	**0.000**
Antifungal agent, *n* (%)	1917 (41.7%)	98 (43.6%)	0.640	256 (51.9%)	**0.000**
Long-term outcome					
Oxygen therapy (days, median [IQR])	3 [0-17]	0 [0-12]	**0.009**	0 [0-7]	**0.000**
Noninvasive ventilation (days, median [IQR])	29 [9–44]	27 [3–46]	0.447	14 [0-43]	**0.000**
Invasive ventilation (days, median [IQR])	23 [8–44]	22 [9–42]	0.972	39 [21–64]	**0.000**
BPD, *n* (%)	3712 (95.7%)	159 (94.1%)	0.397	298 (99.7%)	**0.001**
Parenteral nutrition (days, median [IQR])	32 [18–51]	42 [28–60]	**0.000**	60 [28–93]	**0.000**
Hospitalization (days, median [IQR])	92 [71–115]	85 [61–114]	0.060	95 [35–140]	0.884

*SGA* small-for-gestational-age, *IVF* In vitro fertilization, *GDM* gestational diabetes mellitus, *PIH* pregnancy induced hypertension, *PROM* premature rupture of membranes, *RDS* respiratory distress syndrome, *PDA* patent ductus arteriosus, *IVH* intraventricular hemorrhage, *BPD* bronchopulmonary dysplasia.

Statistically significant *p* < 0.05 values are in bold.

The clinical morbidities significantly associated with mNEC were air leak syndrome (*p* = 0.030), pulmonary hypertension (*p* = 0.020), RDS (*p* = 0.018), hypotension (*p* = 0.001), IVH (≥grade 2, *p* = 0.002), and late-onset sepsis (*p* < 0.001). The neonatal factors significantly correlated with sNEC were lower GA (*p* < 0.001), lower BW (*p* < 0.001), SGA infants (*p* = 0.010), delivery mode (cesarean section, *p* = 0.022), and low Apgar score at 5 min (*p* < 0.001). The clinical morbidities significantly associated with sNEC were air leak syndrome (*p* < 0.001), massive pulmonary hemorrhage (*p* < 0.001), pulmonary hypertension (*p* < 0.001), clinically symptomatic PDA (*p* = 0.024), hypotension (*p* < 0.001), IVH (≥grade 2, *p* < 0.001), late-onset sepsis (*p* < 0.001), and use of antifungal agents (*p* < 0.001).

In infants with mNEC, duration for oxygen therapy (0 [0–12] d vs 3 [0–17] d) was shorter compared to that in infants without NEC, while duration for parenteral nutrition (42 [28–60] d vs 32 [18–51] d) was longer. In infants with sNEC, duration for oxygen therapy (0 [0–7] d vs 3 [0–17] d) and noninvasive ventilation (14 [0–43] d vs 29 [9–44] d) was shorter compared to infants without NEC. However, duration for invasive ventilation (39 [21–64] d vs 23 [8–44] d) and parenteral nutrition (60 [28–93] d vs 32 [18–51] d) was longer in sNEC group. Additionally, incidence of BPD (99.7% vs 95.7%) was higher in infants with sNEC.

### Characteristics of EPIs with NEC matched in a 1:5 ratio based on GA

GA is significantly associated with the development of NEC; therefore, GA was used as a covariate in the matching process. Logistic regression was used for estimating propensity scores, and a 1:5 nearest neighbor propensity score matching was conducted. The characteristics of the EPIs with and without NEC after matching process are presented in Table 2.

**Table 2:** Characteristics of EPIs with NEC matched in a 1:5 ratio based on GA.

	Control group (*n* = 1125)	mNEC group (*n* = 225)	*p*-value	Control group (*n* = 2465)	sNEC group (*n* = 493)	*p*-value
Neonatal factor						
Gestational age (weeks, median [IQR])	26.1 [24.9–27.1]	26.1 [24.9–27.1]	0.881	25.3 [24.4–26.4]	25.1 [24.3–26.3]	0.072
Birth weight (gram, median [IQR])	840 [698–989]	820 [670–990]	0.411	770 [650–990]	720 [620–870]	**0.001**
Male, *n* (%)	575 (51.1%)	128 (56.9%)	0.131	1298 (52.7%)	275 (55.9%)	0.206
SGA, *n* (%)	90 (8.0%)	21 (9.3%)	0.595	189 (7.7%)	52 (10.5%)	**0.041**
Multiple birth, *n* (%)	327 (29.1%)	63 (28.0%)	0.809	797 (32.3%)	168 (34.1%)	0.483
Cesarean section, *n* (%)	821 (73.0%)	174 (77.3%)	0.203	1751 (71.0%)	338 (68.6%)	0.295
Low Apgar score at 1 min, *n* (%)	1103 (98.7%)	222 (99.1%)	0.825	2323 (95.0%)	461 (95.1%)	1.000
Low Apgar score at 5 min, *n* (%)	564 (50.4%)	113 (50.4%)	1.000	1267 (51.8%)	284 (58.6%)	**0.007**
Resuscitation at delivery, *n* (%)	1098 (97.9%)	222 (99.1%)	0.367	2429 (99.0%)	482 (99.0%)	1.000
Maternal factor						
Maternal age (median [IQR])	34 [31–36]	33 [31–36]	0.595	33 [31–36]	34 [31–36]	0.389
IVF, *n* (%)	266 (23.6%)	56 (24.9%)	0.753	682 (27.7%)	123 (24.9%)	0.237
GDM, *n* (%)	87 (7.7%)	20 (8.9%)	0.652	158 (6.4%)	37 (7.5%)	0.426
PIH, *n* (%)	149 (13.2%)	28 (12.4%)	0.829	255 (10.3%)	58 (11.8%)	0.392
Chorioamnionitis, *n* (%)	491 (49.2%)	92 (45.5%)	0.377	1183 (55.1%)	189 (47.5%)	**0.006**
PROM, *n* (%)	487 (43.8%)	112 (49.8%)	0.113	1119 (45.8%)	213 (43.9%)	0.481
Abnormal amniotic fluid volume, *n* (%)	171 (16.3%)	29 (14.1%)	0.488	377 (16.7%)	74 (17.1%)	0.903
Complete antenatal steroid, *n* (%)	554 (58.7%)	104 (55.3%)	0.439	1209 (58.2%)	205 (51.6%)	**0.018**
Antenatal antibiotics, *n* (%)	234 (71.6%)	49 (75.4%)	0.633	559 (73.6%)	88 (66.2%)	0.098
Clinical morbidities						
Congenital infection, *n* (%)	9 (0.8%)	5 (2.2%)	0.118	42 (1.7%)	9 (1.8%)	1.000
Air leak syndrome, *n* (%)	94 (8.4%)	26 (11.6%)	0.158	212 (8.6%)	62 (12.6%)	**0.007**
Massive pulmonary hemorrhage, *n* (%)	109 (9.7%)	28 (12.4%)	0.259	254 (10.3%)	79 (16.0%)	**0.000**
Pulmonary hypertension, *n* (%)	182 (16.2%)	50 (22.2%)	**0.036**	507 (20.6%)	132 (26.8%)	**0.003**
RDS, *n* (%)	1092 (97.1%)	212 (94.2%)	0.052	2403 (97.5%)	483 (98.0%)	0.631
Surfactant use, *n* (%)	1102 (98.0%)	217 (96.4%)	0.255	2427 (98.5%)	483 (98.0%)	0.558
Symptomatic PDA, *n* (%)	593 (52.7%)	117 (52.0%)	0.903	1349 (54.7%)	278 (56.4%)	0.530
Hypotension, *n* (%)	501 (44.5%)	119 (52.9%)	**0.026**	1214 (49.2%)	323 (65.5%)	**0.000**
IVH (≥grade2), *n* (%)	368 (32.7%)	91 (40.4%)	**0.031**	887 (36.0%)	245 (49.7%)	**0.000**
Early-onset sepsis, *n* (%)	25 (2.2%)	6 (2.7%)	0.871	104 (4.2%)	20 (4.1%)	0.967
Late-onset sepsis, *n* (%)	330 (29.3%)	97 (43.1%)	**0.000**	817 (33.1%)	248 (50.3%)	**0.000**
Antifungal agent, *n* (%)	487 (43.3%)	98 (43.6%)	1.000	1162 (47.1%)	256 (51.9%)	0.058
Long-term outcome						
Oxygen therapy (days, median [IQR])	2 [0-16]	0 [0-12]	**0.041**	2 [0-17]	0 [0-7]	**0.000**
Noninvasive ventilation (days, median [IQR])	28 [7–44]	27 [3–46]	0.758	28 [6–45]	14 [0-43]	**0.000**
Invasive ventilation (days, median [IQR])	23 [9–47]	22 [9–42]	0.431	29 [12–50]	39 [21–64]	**0.000**
BPD, *n* (%)	895 (96.2%)	159 (94.1%)	0.276	1904 (98.0%)	298 (99.7%)	0.072
Parenteral nutrition (days, median [IQR])	32 [18–52]	42 [28–60]	**0.000**	34 [20–55]	60 [28–93]	**0.000**
Hospitalization (days, median [IQR])	93 [70–117]	85 [61–114]	0.069	98 [69–123]	95 [35–140]	0.510

*SGA* small-for-gestational-age, *IVF* In vitro fertilization, *GDM* gestational diabetes mellitus, *PIH* pregnancy induced hypertension, *PROM* premature rupture of membranes, *RDS* respiratory distress syndrome, *PDA* patent ductus arteriosus, *IVH* intraventricular hemorrhage, *BPD* bronchopulmonary dysplasia.

Statistically significant *p* < 0.05 values are in bold.

The clinical morbidities significantly associated with mNEC in matched groups (control, 1,125 vs mNEC, 225) included pulmonary hypertension (*p* = 0.036), hypotension (*p* = 0.026), IVH (≥grade 2, *p* = 0.031), and late-onset sepsis (*p* < 0.001). Incidence of air leak syndrome and RDS was not significant in the matched group. The neonatal factors significantly correlated with sNEC in matched groups (control, 2465 vs sNEC, 493) were lower BW (*p* = 0.001) and SGA (*p* = 0.041). The matched sNEC group exhibited a significantly higher rate of complete administration of antenatal steroids (*p* = 0.018) compared to control group. The clinical morbidities significantly associated with sNEC were air leak syndrome (*p* = 0.007), massive pulmonary hemorrhage (*p* < 0.001), pulmonary hypertension (*p* = 0.003), hypotension (*p* < 0.001), IVH (≥grade 2, *p* < 0.001), and late-onset sepsis (*p* < 0.001). After matching based on GA, incidence of clinically symptomatic PDA and BPD, use of antifungal agent, delivery mode, and low Apgar score at 5 min were not significantly associated with sNEC.

Among matched infants with mNEC, duration for oxygen therapy (0 [0–12] d vs 2 [0–16] d) was shorter compared to that in infants without NEC, while duration for parenteral nutrition (42 [28–60] d vs 32 [18–52] d) was longer. In matched infants with sNEC, duration for oxygen therapy (0 [0–7] d vs 2 [0–17] d) and noninvasive ventilation (14 [0–43] d vs 28 [6–45] d) was shorter compared to infants without NEC. However, duration for invasive ventilation (39 [21–64] d vs 29 [12–50] d) and parenteral nutrition (60 [28–93] d vs 34 [20–55] d) was longer in matched sNEC group.

### Risk factor analysis for NEC in matched EPIs

**Fig. 2:**
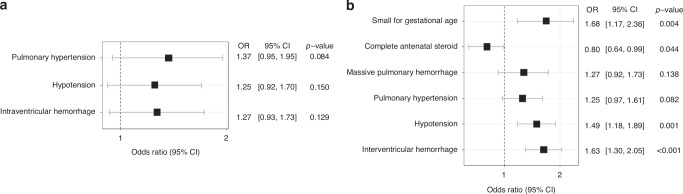
Odds ratio for NEC in EPIs. Backward stepwise logistic regression was conducted to analyze risk factors associated with NEC in EPIs. **a** Plot of the odds ratios for mNEC. Pulmonary hypertension, hypotension, and IVH are included in the final model. There was no risk factor identified to be significantly associated with the development of mNEC. **b** Plot of the odds ratios for sNEC. SGA, complete administration of antenatal steroid, massive pulmonary hemorrhage, pulmonary hypertension, hypotension, and IVH are included in the final model. SGA, hypotension, and IVH significantly increased the risk of sNEC with odds ratio of 1.68, 1.49, and 1.63, respectively. Antenatal steroid was a protective factor for sNEC with odds ratio of 0.80. NEC necrotizing enterocolitis, EPI extremely preterm infants, IVH intraventricular hemorrhage, mNEC medically treated NEC, sNEC surgically treated NEC, SGA small for gestational age, OR odds ratio, CI confidence interval.

Clinical characteristics of infants with NEC was assessed to identify factors contributing to the development of NEC in EPIs (Table 2). For the risk factor analysis, a backward stepwise logistic regression was conducted (Fig. 2). In cases of mNEC, pulmonary hypertension, hypotension, and IVH were included in the final model. No significant factors were associated with the increased risk of mNEC. In risk factor analysis for sNEC, SGA, complete administration of antenatal steroid, massive pulmonary hemorrhage, pulmonary hypertension, hypotension, and IVH were included in the final model. As a result, infants who were SGA, experienced hypotension, and IVH were found to be significantly associated with an increased risk of developing sNEC. Specifically, the odds ratio (OR) for SGA was 1.68 (95% confidence interval [CI], 1.17–2.36; *p* = 0.004), for hypotension was 1.49 (95% CI, 1.18–1.89; *p* = 0.001), and for IVH was 1.63 (95% CI, 1.30–2.05; *p* < 0.001). Complete administration of antenatal steroids was identified as a protective factor for sNEC (OR 0.80, 95% CI, 0.64–0.99; *p* = 0.044). Late-onset sepsis was significantly different between both mNEC and sNEC groups, compared to the control group. However, it was excluded from the risk factor analysis due to incomplete clinical record of NEC occurrence date.

## Discussion

In previous studies, the incidence of NEC increased as GA decreased, and EPIs showed a higher incidence of NEC.^1,5,22,23^ In a previous study, we demonstrated that the incidence of NEC was strongly associated with low GA, and EPIs were more likely to undergo surgical intervention rather than medical treatment for NEC.^18^ Considering the adverse effects of sNEC on the long-term prognosis of preterm infants, identification of the predictive factors for sNEC among EPIs is important. EPIs with sNEC included in this study required a longer duration of invasive ventilation, and prolonged use of parenteral nutrition compared to those without sNEC while requiring shorter durations of oxygen therapy and noninvasive ventilation. Additionally, they exhibited a higher incidence of BPD, which is likely associated with the prolonged respiratory support. Moreover, they experienced more frequent episodes of late-onset sepsis. These findings suggest that NEC may contribute to the risk factors for sepsis, including prolonged use of central catheters and parenteral nutrition, and extended duration of ventilation. Interestingly, no statistically significant difference was observed in the length of hospital stay.

In the risk factor analysis conducted after matching with GA as a covariate, SGA, hypotension, and IVH were identified as independent risk factors for sNEC in EPIs. Previous studies have reported that infants with lower BW are more likely to undergo surgery for NEC.^8,15^ Hull et al. reported a large cohort study (Vermont Oxford Network) conducted in the United States and found that the probability of surgical treatment for NEC decreased as BW increased, and the odds of surgery decreased by 5% for each 100 g increase in BW.^8^ Contrary to other studies, multivariable analysis in our study, which was conducted by matching for GA, confirmed that lower BW itself was not associated with an increased risk of sNEC. Considering the correlation between GA and BW of EPIs included in this study, this result may have been influenced by the fact that BW tends to decrease as GA decreases (*p* < 0.001). However, the odds of sNEC were significantly correlated with SGA (OR 1.68, 95% CI, 1.17–2.36; *p* = 0.004). In previous studies, SGA infants showed a higher risk of developing NEC compared to appropriate for gestational age (AGA) infants.^24–26^ In our study with EPIs, we confirmed that SGA significantly contributes to the occurrence of sNEC, while its effect on medically treatable NEC was insignificant.

Hypotension (OR 1.49, 95% CI, 1.18–1.89; *p* = 0.001), and IVH (OR 1.63, 95% CI, 1.30–2.05; *p* < 0.001) were independent risk factors for sNEC in this study. The prevalence of hypotension in the entire cohort of EPIs was 44.6%, while it was higher in the sNEC group (65.5%). Hypotension being a risk factor for NEC has been reported in previous studies. Data from the Canadian Neonatal Network confirmed a relationship between the treatment of hypotension and the occurrence of NEC.^27^ This association between hypotension and NEC was further supported by findings from a systematic review on risk factors for NEC^28^ and the study which reported higher prevalence of surgery for NEC in the group with a higher incidence of grade 4 IVH assessed by early postnatal brain ultrasound.^29^ Our results may explain hypoxic-ischemic injury as a pathogenesis of NEC. The role of hypoxia and ischemia as primary contributors to the development of NEC has been questioned; however, it probably has a secondary role.^30^ Impaired intestinal perfusion and ischemia-reperfusion can play role in NEC development.^31–34^ Hypoxia and ischemia in immature intestinal circulation are related to the production of vascular regulators, such as nitric oxide and endothelin, which probably leads to NEC.^5,6,35,36^ In addition, the connection between superior mesenteric artery blood flow and the risk of NEC has been revealed through a recent study.^37,38^ Therefore, our results suggest the possibility that hypotension or IVH may affect intestinal circulation due to hemodynamic instability,^39^ which is potentially related to the development of sNEC. However, it should be considered that IVH may not be the primary cause of sNEC. IVH could arise as a complication of NEC itself or from surgical interventions. Jen et al. demonstrated that sNEC increases the risk of IVH progression.^40^ On the contrary, another study reported a limited association between episodes of NEC and the occurrence or progression of IVH.^41^

This study supports previous study findings that antenatal steroids decrease the risk of NEC. The results of the NICHD Neonatal Research Network study, conducted on infants born between 22 and 25 weeks of GA, showed the incidence of death or NEC was lower in infants whose mothers received antenatal steroids, even at the GA of 22 weeks.^42^ In other studies, with preterm infants born < 35 weeks of GA, the administration of antenatal steroids was also found to decrease the occurrence of NEC.^43,44^ The odds ratio for administration of antenatal steroids in relation to sNEC was 0.80 (95% CI, 0.64-0.99; p = 0.044) in this study. Animal studies in a rat model demonstrated that antenatal steroids have a protective effect on the incidence and progression of NEC.^45,46^ The administration of antenatal steroids promotes the maturation and maintenance of the intestinal barrier and reduces systemic inflammation.

The present study had some limitations. First, we were unable to evaluate the temporal relationships between NEC and associated clinical events due to a lack of information on the timing of clinical events. In addition, the analysis was limited because data on factors believed to be related to the development of NEC, such as enteral feeding type and timing of full enteral feeding, were not included. However, the study analyzed data from a nationwide cohort (KNN) of 77 NICUs in Korea to identify risk factors related to sNEC in the EPI group, which showed a higher incidence of sNEC compared to other groups. The use of a large nationwide database is a key strength of this study. We focused on sNEC for the objectivity of NEC diagnosis in this study. Diagnosis of NEC is based on clinical signs, radiographical findings, surgical findings, or autopsy.^47^ However, various diseases and conditions need to be differentiated from NEC.^5,6^ Nonspecific clinical findings of NEC, such as abdominal distension, are common symptoms in VLBW infants and can also be caused by other diseases. In addition, there are cases in which radiological findings of NEC are not presented.^48^ Medical management is performed even when NEC is only suspected. Therefore, given the subjective nature of mNEC diagnosis, greater objectivity can be provided by focusing on sNEC.

NEC is the serious gastrointestinal disease and has poor outcomes, especially in EPIs. Our study may support the clinician to identify infants with increased risk for sNEC, potentially leading to earlier diagnosis or surgical intervention. We identified SGA, hypotension within 7 d after birth, and IVH as independent predictive risk factors for sNEC, and complete administration of antenatal steroids as a protective factor in GA-matched EPIs. EPIs with SGA, low blood pressure, and IVH require close attention and monitoring for any signs of surgical indications of NEC.

## Data Availability

The data that support the findings of this study are available from the Korean Neonatal Network and the Korea Centers for Disease Control and Prevention, but restrictions apply to the availability of these data, which were used under license for the current study, and so are not publicly available. Data are however available from the authors upon reasonable request and with permission of the Korean Neonatal Network and the Korea Centers for Disease Control and Prevention.
